# Cutaneous Metastatic Breast Cancer Masked by Hidradenitis Suppurativa

**DOI:** 10.7759/cureus.12862

**Published:** 2021-01-22

**Authors:** Rena A Cohen-Kurzrock, Ryan R Riahi

**Affiliations:** 1 Health Administration, Louisiana State University of Shreveport, Shreveport, USA; 2 Dermatology, DermSurgery Associates, Sugar Land, USA

**Keywords:** adenocarcinoma, breast, cancer, carcinoma, cutaneous, hidradenitis, malignancy, masked, metastasis, suppurativa

## Abstract

The appearance of cutaneous metastases from breast carcinoma is variable and can present as nodules or inflammation of the skin, mimicking benign skin conditions. In addition, the skin lesions may be the initial presentation of unsuspected visceral malignancy or the site of either persistence or recurrence of metastatic disease in an oncology patient with a history of a solid tumor. The features of a woman with metastatic breast cancer that presented as a nodule that was masked by her concurrent, new-onset, hidradenitis suppurativa are reported. The diagnosis was suspected when the skin nodule persisted after her hidradenitis suppurativa improved; the diagnosis of cutaneous metastasis was confirmed with a skin biopsy. Occult breast cancer (primary or recurrent disease) may be masked by an inflammatory condition, such as hidradenitis suppurativa. Therefore, if a primary dermatologic condition does not appropriately respond to therapy, pathologic evaluation may be warranted to exclude the possibility of another disease, such as cutaneous metastases from underlying visceral cancer.

## Introduction

Breast carcinoma is the second most common internal malignancy in women and the most common solid organ tumor to metastasize to the skin [1-4]. The development of cutaneous metastasis from an internal malignancy is uncommon [4]. However, albeit rare, occult cancer can present as or be masked by an inflammatory condition, such as hidradenitis suppurativa [4].

Hidradenitis suppurativa is a chronic inflammatory skin condition that can affect the quality of a person’s life. It is common amongst young adults and clinically presents with recurrent, inflammatory abscesses, fistulas, nodules, and sinuses. Hidradenitis suppurativa can involve the axilla, breasts, buttocks, and groin. The condition may be associated with significant morbidity; associated conditions in patients with hidradenitis suppurativa include depression, diabetes mellitus, and Crohn’s disease [5,6].

Cutaneous metastasis from breast carcinoma can mimic the clinical presentation of other conditions [7-12]. A 30-year-old woman with hidradenitis suppurativa of the axilla, breast, and groin was noted to have a firm nodule on her right breast. When her hidradenitis suppurativa responded to treatment-yet the nodule persisted-malignancy was considered and a four-millimeter punch biopsy of the nodule was performed. The biopsy showed adenocarcinoma and the subsequent workup confirmed the diagnosis of breast cancer with metastasis to the skin. Occult breast cancer (primary or recurrent disease) can either be masked by or mimic inflammatory skin conditions. Malignancy should be entertained and further workup considered when a primary dermatologic condition does not respond to appropriate medical therapy.

## Case presentation

A 30-year-old woman presented for evaluation of draining boils and sores that were located between and on the breasts, both axillae and the groin for seven months. She had previously been treated by another clinician with benzoyl peroxide five percent body wash with mild benefit. She was not taking any other medications and she had no other medical conditions. Her family history was significant for breast cancer in a cousin and her mother.

**Figure 1:**
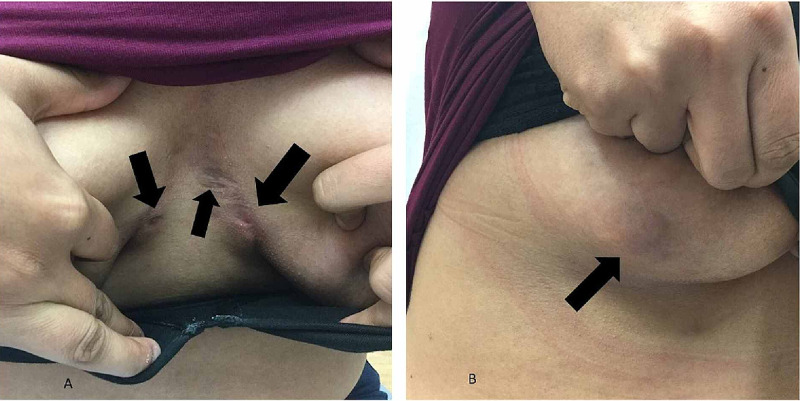
Cutaneous hidradenitis suppurativa masking breast cancer A 30-year-old woman presented with erythematous nodules and draining sinus tracts (black arrows) of hidradenitis suppurativa on and in between the breasts (A). A four-centimeter red nodule (black arrow) on the right breast was initially interpreted as hidradenitis suppurativa (B).

Cutaneous examination revealed draining sinus tracts between the breasts and in the axillae. Scars were observed on the breast area and an indurated, four-centimeter nodule was appreciated on the right breast (Figure 1). Based on her clinical history and skin findings, a diagnosis of hidradenitis suppurativa was established and daily therapy with oral minocycline (100 milligrams), topical benzoyl peroxide five percent wash, and topical clindamycin one percent solution was initiated.

**Figure 2:**
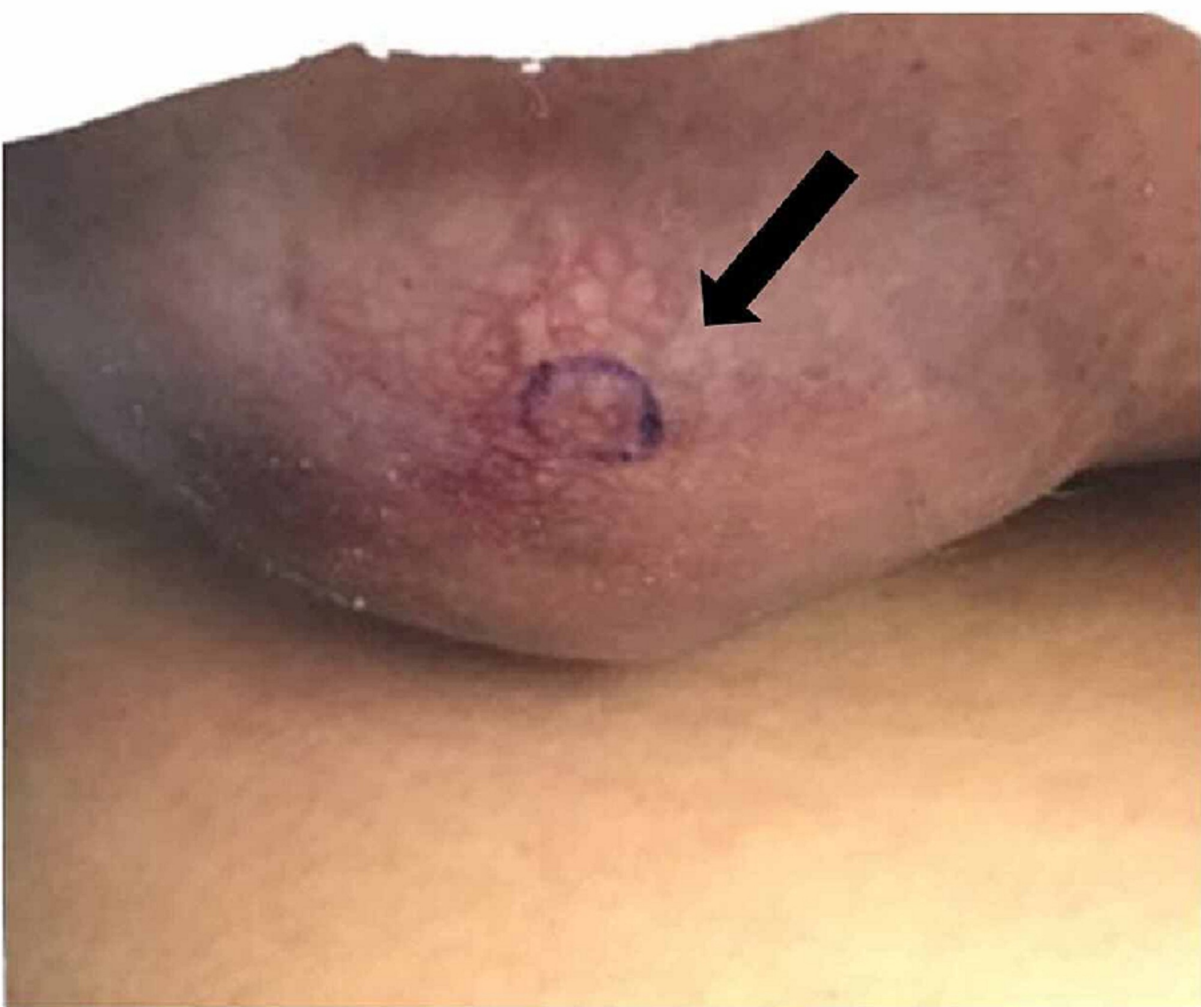
Metastatic adenocarcinoma of the right breast presenting as an enlarging red nodule that mimic hidradenitis suppurativa in a 30-year-old woman A persistent enlarging red nodule (black arrow) on the right breast that did not respond to oral and topical therapy for hidradenitis suppurativa. A punch biopsy (taken from the area within the purple circle) demonstrated an adenocarcinoma of the breast that had metastasized to the skin.

She returned for a follow-up evaluation three months after her initial visit. She thought that her condition was well controlled. However, cutaneous examination demonstrated a persistent nodule on the right breast that appeared larger than it was on her initial visit (Figure 2).

Malignancy was considered in the different diagnosis of the right breast nodule. A four-millimeter punch biopsy was performed. The microscopic evaluation demonstrated an adenocarcinoma of the breast. The patient was referred to an oncologist. Additional workup confirmed the diagnosis of metastatic breast cancer. She completed a surgical resection of the right breast and is currently undergoing chemotherapy.

## Discussion

Visceral metastases to the skin are seldom the presenting sign of an undiagnosed malignancy or the site of recurrence in an oncology patient with a history of a solid tumor. Cutaneous metastases can have several morphologies; therefore, in a patient in whom metastatic cancer is unsuspected, there can be a delay in diagnosis and treatment [3,4,13]. Metastases to the skin tend to occur close to the proximity of the primary tumor; however, any location can be involved since metastatic tumors may spread to the skin either by direct extension from an underlying malignancy or by vascular and lymphatic channels [1]. 

Cutaneous breast carcinoma can manifest with a variety of appearances. Nodules are the most common presentation of cutaneous metastases [3,7]. In addition to an inflammatory condition such as hidradenitis suppurativa, occult breast cancer can mimic dermatologic conditions (alopecia, dermatitis, periorbital edema, and scleroderma), bacterial (acute paronychia and cellulitis) or viral (varicella-zoster) infections, or vascular lesions (lymphangioma circumscriptum, palpable purpura, and pyogenic granuloma) [1,7-12]. The reported patient’s clinical features included draining nodules and sinus tracts not only on but also in between her breasts. Indeed, her hidradenitis suppurativa masked the cutaneous metastasis from the underlying breast adenocarcinoma.

When conditions of the hair, skin, and nails do not respond to appropriate medical therapy, additional evaluation should be considered to exclude the possibility of malignancy. A 67-year-old woman with biopsy-proven onychomycosis of a single fingernail did not respond to appropriate anti-fungal therapy; therefore, a nail avulsion was performed and the biopsy of the underlying friable red nodule has established the diagnosis of amelanotic melanoma [11,12]. Similar to the reported woman, Lookingbill et al. described a patient who presented with a fluctuant and purulent draining perianal abscess that was initially diagnosed as hidradenitis suppurativa; a subsequent biopsy established the diagnosis of metastatic rectal mucinous adenocarcinoma [4].

## Conclusions

Cutaneous metastases are rarely the initial presentation of an unsuspected internal malignancy. Primary or recurrent occult breast cancer may present as or be masked by an inflammatory skin condition. Our patient had hidradenitis suppurativa; however, one of the nodules that was present at the initial visit did not completely resolve with appropriate medical therapy. A biopsy of this persistent nodule on her right breast established the additional diagnosis of a cutaneous metastasis as the initial presentation of her unsuspected breast adenocarcinoma. Therefore, if a dermatologic condition or a skin lesion does not respond to appropriate medical therapy, additional evaluation--including a biopsy--should be considered.
